# Impact of urban birth and upbringing on expression of psychosis in a Chinese undergraduate population

**DOI:** 10.1186/s12888-021-03475-w

**Published:** 2021-10-09

**Authors:** Jeremy W. Coid, Yamin Zhang, Huan Sun, Hua Yu, Wei Wei, Xiaojing Li, Qiuyue Lv, Wanjie Tang, Qiang Wang, Wei Deng, Wanjun Guo, Liansheng Zhao, Xiaohong Ma, Yajing Meng, Mingli Li, Huiyao Wang, Ting Chen, Tao Li

**Affiliations:** 1 Mental Health Center and Psychiatric Laboratory, the State Key Laboratory of Biotherapy, West China Hospital of Sichuan University, No. 28 Dianxin South street, Chengdu, 610041 Sichuan China; 2 Institute of Emergency Management and Post-disaster Reconstruction, Sichuan University, Chengdu, Sichuan China; 3 Centre for Psychological Educational and Consultation, Sichuan University, Chengdu, Sichuan China; 4 Hangzhou Seventh People’s Hospital, Affiliated Mental Health Center, Zhejiang University School of Medicine, Hangzhou, Zhejiang China

**Keywords:** Urbanicity, Schizophrenia, Psychotic-like experiences, Etiology

## Abstract

**Background:**

Urban birth and upbringing show consistent associations with psychotic illness but the key urban exposures remain unknown. Associations with psychotic-like experiences (PEs) are inconsistent. These could be confounded by common mental disorders associated with PEs. Furthermore, associations between PEs and urban exposures may not extrapolate to psychotic disorders such as schizophrenia.

**Methods:**

Annual cross-sectional surveys among first year Chinese undergraduates 2014–2019 (*n* = 47,004). Self-reported, hierarchical categorisation of psychosis: from psychoticism, paranoid ideation, schizotypal symptoms, nuclear syndrome using SCL-90-R, to clinical diagnosis of schizophrenia. Depressive symptoms using PHQ 9. Dissociative symptoms and posttraumatic stress disorder (PTSD) measured using PCL-C. Etiological factors of family history and childhood disadvantage. We studied effects of urban birth, urban living and critical times of exposure in childhood on psychosis phenotypes.

**Results:**

Associations with urbanicity were found only after adjustments for depression. Urban birth was associated with paranoia (AOR 1.34, 1.18–1.53), schizotypal symptoms (AOR 1.59, 1.29–1.96), and schizophrenia (AOR 2.07, 1.10–3.87). The same phenotypes showed associations with urban residence > 10 years. Only schizophrenia showed an association with urban exposure birth-3 years (AOR 7.01, 1.90–25.86). Child maltreatment was associated with both psychosis and depression. Urbanicity measured across the total sample did not show any associations with demography, family history of psychosis, or child maltreatment. Sensitivity analysis additionally adjusting for dissociative symptoms and PTSD showed the same pattern of findings.

**Conclusions:**

Urban birth and urban living showed a hierarchical pattern of increasing associations from paranoid ideation to schizotypal disorder to schizophrenia, confirming that associations for psychotic experiences could be extrapolated to schizophrenia, but only after adjusting for confounding from depression, dissociative symptoms and PTSD. Several etiological factors were the same for psychosis and depression. Future studies of PEs should adjust for confounding from common mental disorders and dissociative symptoms. Effects of urbanicity on psychosis were not explained by demography, family history of mental disorder, or child maltreatment.

**Supplementary Information:**

The online version contains supplementary material available at 10.1186/s12888-021-03475-w.

## Background

Being born and growing up in an urban environment are among the most consistently reported associations with schizophrenia and other forms of psychosis [1–5]. Meta-analysis has shown a pooled OR for psychosis of 2.39 (95% CI 1.62–3.51) compared to rural environments [6]. However, there is still no consensus on what the key exposures are in the urban environment which increase psychosis risk. Possibilities include stress caused by physical and social environmental factors, gene-environment interactions, environment-environment interactions, and biological effects of factors such as pollution and infections. Timing of exposure is important because it may give some indication of what the key exposures are. For example, whether the exposure occurs before birth, during critical periods of brain development particularly infancy, or cumulative effects of total time of exposure from birth to early adulthood. Urban birth could implicate exposures in utero such as maternal infections or birth trauma; exposures in infancy could also include infections, environmental pollution, or quality of parental care on the developing brain; prolonged exposure from birth to early adulthood could implicate cumulative effects of each of these and a range of additional factors, environment-environment, or gene-environment interactions. A further question is whether these urban exposures impact on the full range of phenotypic expression of psychosis, from psychotic-like experiences (PEs) to schizophrenia.

China is an important location for studying urbanicity because it has undergone the most rapid process of urbanization of any country from the mid-twentieth century onwards. Surveys have found consistently higher prevalence of schizophrenia compared to other regions worldwide [7]. This is thought to be increasing [8] but is not confirmed. In the past, most prevalence studies in China showed highest rates of schizophrenia and most other mental disorders in rural rather than urban areas [9, 10] associated with poverty and inequalities in access to healthcare [11]. More recently, this pattern has changed with prevalence studies corresponding increasingly to western countries [12] and most probably corresponding to population shift and encountering new exposures in an urban environment. Studies of PEs have shown inconsistency. Urban birth was associated with high but not low levels of PEs in a representative sample of young adult men, particularly among those currently living in an urban environment [13]. In contrast, a study of undergraduates found rural birth/upbringing associated with PEs, together with female gender and childhood trauma [14]. However, this study did not adjust for symptoms of common mental disorders.

### Does depression confound the urbanicity-psychosis association?

One explanation for inconsistency could be that PEs can be the outcome of severe depression, anxiety, and dissociative symptoms as well as being on a continuum with psychotic illness such as schizophrenia [15–20]. PEs modify clinical and functional severity of depression resulting in poorer course and outcome [15, 20]. Exposure of non-psychotic conditions such as depression to genetic and environmental risk factors is associated with more severe, non-psychotic psychopathology, which in turn is associated with greater probability of psychosis [21, 22]. Furthermore, it has been argued that individuals with PEs are more likely to develop mood disorders rather than psychotic disorder [17], although community studies have not consistently shown this.

If rural birth were associated with risk factors more strongly associated with co-occurring depression, inconsistency in findings for associations with PEs may be determined by whether studies controlled for confounding by depression when investigating associations between PEs and urbanicity.

The aims of this study were to investigate 1) associations between level of urbanicity of place of birth and the outcome of different phenotypic expression of psychosis measured using PEs and a clinical measure of schizophrenia, 2) associations between length of exposure to an urban environment and psychosis, 3) associations between critical timing of exposures to the urban environment between birth and 15 years, and 4) whether demographic, family history of mental disorder, and child maltreatment were associated with urbanicity to account for these associations. For each of these aims, we investigated associations before and after adjustments for depressive symptoms.

## Methods

### Participants

The Sichuan University Students Study is an ongoing investigation into mental health problems associated with stress factors in student life, risk factors preceding university entry, and their impact on academic performance and mental health. All freshmen are invited annually to complete a questionnaire on-line, with a follow-up subsample at 1 year. The first year cross-sectional study sample was used for this investigation and included male and female respondents, 2014–2019. After excluding those who gave incomplete information, 47,004 were included, an 84.1% response rate.

### Measures

#### Urbanicity

All participants were asked about their birthplace. We used a six-level rating method previously used by Mortensen and colleagues [23] ranking from countryside, township, county-level city, prefecture-level city, to provincial capital city and municipality in China. In this study, we combined levels 1 and 2 (most rural), 3 and 4, and 5 and 6 (most urban).

Participants were asked about their age at time of any moves between the different levels rural-urban during their upbringing, from birth to 15 years. To assess the cumulative exposure of an urban environment, we calculated total time spent at level 3 until age 15 years and compared them with participants who had never spent time at level 3 during their upbringing.

To assess the effects of critical timings from birth to 15 years old of urban level 3 residence, we divided the 15 years into three periods of 5 years, from birth to 5 years, 5 to 10 years, 10 to15 years. For each period, we divided subjects into three groups of 0 years, 1 to 3 years, 4 to 5 years, and compared the latter two to participants who never spent time at level 3.

#### Psychosis phenotypes

We created categorical psychosis phenotypes as a hierarchy of severity from psychotic experiences measured by psychoticism and paranoid ideation, psychotic symptoms measured by schizotypal symptoms and nuclear syndrome symptoms, and finally a clinical diagnosis of schizophrenia. We used the Symptom Checklist-90-Revised (SCL-90-R) [24] to measure four categorical phenotypes of psychosis. It has shown reliability among both the Chinese general population [25] and university students [26]. Firstly, we used the two symptom dimensions relevant to psychosis, including the 10 items in the psychoticism and 6 items in the paranoia subscales to create categories using mean plus 2 times standard deviation (SD), where a student with scores above this cutoff were considered to present with categories of psychoticism and paranoid ideation. We next created two additional categorical psychosis measures showing closer similarity to clinical phenotypes used in clinical practice. These were based on a previously developed SCL-R-90 sub-scale [27]. Instead of using a cut-off based on continuous scores, we re-coded SCL-R-90 items as symptoms, present when scoring 2 (“moderate” self-reported severity) or above for (i) Schizotypal symptoms (including items 8, 18, 43, 68, 76, 77, 83 and 88, shown in supplementary material) were present when there were ≥ 5 of 8 items rated ≥2. (ii) Schizophrenia Nuclear Syndrome was present when there is ≥3 of 4 items rated ≥2 (including items 7, 16, 35 and 62, shown in supplementary material).

Finally, we asked participants if they had ever consulted a medical practitioner and received a clinical diagnosis of schizophrenia.

#### Depression and dissociative symptoms

The Patient Health Questionnaire-9 (PHQ-9) Depression module of the Primary Care Evaluation of Mental Disorders (PRIME-MD) diagnostic instrument for common mental disorders measured Depressive symptoms over the past 2 weeks [28]. Depressive symptoms were used to adjust for confounding. The posttraumatic stress disorder (PTSD) CheckList-Civilian Version (PCL-C) measured dissociative and PTSD symptoms [29]. This measure was introduced into the annual student surveys in 2016 which meant that the sample size was smaller for students completing this and depression measures (see Supplementary file).

#### Etiological risk factors

Participants were asked if first degree relatives had been diagnosed with severe (psychotic or non-psychotic) mental disorders.

Participants self-reported childhood adversities using Childhood Section of the Chinese World Mental Health Initiative Composite International Diagnostic Interview [30, 31], including loss of parent through divorce or death, experience of physical, sexual abuse, or neglect before 16.

### Statistical analysis

We used logistic regression to examine the association between urban exposures and 5 binary psychosis phenotypes. Three categories of urban exposures were included, urban birth, urban upbringing for the first 15 years, and urban upbringing for every 5 years. Logistic regression is also used to assess the co-occurrence of etiological risk factors and their association with psychosis phenotypes. Tables were adjusted for age, sex (and PHQ9 score). Results were presented with odds ratios (ORs) and 95% confidence interval (CI). Statistical significance was set at α = 0.05. All analyses were carried out using R Version 3.3.2. We carried out a sensitivity analysis using a smaller subsample to test the effects on our findings of adding PTSD and dissociative symptoms to our adjustments for depression (see Supplementary file).

## Results

Mean age of the student population was 18.19 years (SD 0.91), 50.1% were male, most Han Chinese (89.8%), with family backgrounds having high or medium level earnings (85.1%).

Table 1 shows associations between the most rural level 1 as reference and other levels of birth-place, with level 3 the most urban, for five categorical phenotypes of psychosis. Psychoticism showed significant negative associations with levels 2 and 3. However, these were attenuated and no longer significant after adjusting for depressive symptoms. Neither paranoid ideation or schizotypal symptoms showed associations with any level before adjustments for depression, but afterwards showed increasing odds of association from levels 2–3. There were no associations with Nuclear syndrome. Clinical diagnosis of schizophrenia showed the highest odds of association among the five phenotypes, but only at urban level 5 and only following adjustment.

**Table 1:** Associations between Rural-Urban levels of Birth place and Psychosis Phenotypes (*N* = 47,004)

	Psychoticism *n* = 1961 (4.2%)		Paranoia *n* = 2572 (5.5%)		Schizotypal symptoms *n* = 867 (1.8%)^a^		Nuclear syndrome *n* = 116 (0.2%)		Diagnosed Schizophrenia *n* = 63 (0.1%)	
	N (%)	OR (95%CI)	N (%)	OR (95%CI)	N (%)	OR (95%CI)	N (%)	OR	N (%)	OR (95%CI)
**Birth Exposure**										
Level 1 (Rural) *n* = 24,479 (52.1%)	1112 (56.7)	Ref (1)	1346 (52.3)	Ref (1)	440 (50.8)	Ref (1)	59 (50.9)	Ref (1)	34 (54.0)	Ref (1)
Level 2 *n* = 15,734 (33.5%)	580 (29.6)	0.80 (0.72–0.89)***	848 (33.0)	0.97 (0.89–1.06)	288 (33.2)	1.00 (0.86–1.16)	38 (32.7)	1.02 (0.67–1.53)	14 (22.2)	0.69 (0.37–1.29)
		0.95 (0.85–1.07)		1.18 (1.07–1.30)***		1.26 (1.07–1.48)**		1.30 (0.85–1.98)		0.73 (0.39–1.38)
Level 3 (Urban) *n* = 6791 (14.4%)	269 (13.7)	0.86 (0.75–0.99)*	378 (14.7)	1.00 (0.89–1.13)	139 (16.0)	1.12 (0.93–1.36)	19 (16.4)	1.18 (0.70–1.98)	15 (23.8)	1.74 (0.94–3.22)
		1.15 (0.99–1.34)		1.34 (1.18–1.53)***		1.59 (1.29–1.96)***		1.71 (1.00–2.93)		2.07 (1.10–3.87)*

Adjusted for age and sex in the first row. Second row for each variable additionally adjusted for PHQ-9 score

^a^Nuclear syndrome excluded from analysis

*P < 0.05, **p < 0.01, ***p < 0.001

Table 2 shows associations between cumulative exposure to an urban environment at level 3 and the psychosis phenotypes. For ease of presentation, associations before adjustment for depression are not shown. However, none of the following findings were observed until adjustment. Participants with no exposure to level 3 between birth and 15 years were reference. Overall length of exposure using a continuous measure over a total of 15 years showed significant associations with schizotypal symptoms and clinical diagnosis of schizophrenia. Cumulative effects of exposure between 6 and 10 years impacted on paranoid ideation, and between 11 and 15 years on Schizotypal symptoms and Schizophrenia. There were no effects of cumulative exposure over time on psychoticism or nuclear syndrome.

**Table 2:** Associations between Length of exposure to an Urban environment and Psychosis Phenotypes (*N* = 47,004)

	Psychoticism *n* = 1961 (4.2%)		Paranoia *n* = 2572 (5.5%)		Schizotypal symptoms *n* = 867 (1.8%)^a^		Nuclear syndrome *n* = 116 (0.2%)		Diagnosed Schizophrenia *n* = 63 (0.1%)	
	N (%)	OR	N (%)	OR (95%CI)	N (%)	OR (95%CI)	N (%)	OR (95%CI)	N (%)	OR (95%CI)
**Urban Exposure**										
Total in 15 years^b^ *n* = 47,004 (100%)	3.6 (6.0)	1.00 (0.99–1.01)	3.9 (6.2)	1.01 (1.00–1.02)	4.4 (6.5)	1.02 (1.01–1.03)***	4.0 (6.4)	1.01 (0.98–1.04)	5.5 (6.8)	1.05 (1.02–1.09)**
0 years (Ref) *n* = 33,550 (71.4%)	1383 (70.5)	Ref (1)	1763 (68.5)	Ref (1)	557 (64.2)	Ref (1)	81 (69.8)	0.50 (0.15–1.63)	36 (57.2)	Ref (1)
1–5 years *n* = 1958 (4.2%)	82 (4.2)	0.82 (0.63–1.06)	105 (4.1)	0.86 (0.69–1.08)	44 (5.1)	1.11 (0.79–1.56)	3 (2.6)	0.61 (0.19–1.98)	1 (1.6)	0.43 (0.06–3.18)
6–10 years *n* = 1598 (3.4%)	81 (4.1)	1.05 (0.80–1.37)	116 (4.5)	1.26 (1.01–1.57)*	35 (4.1)	1.06 (0.73–1.55)	3 (2.6)	1.30 (0.84–1.24)	4 (6.3)	2.46 (0.85–7.12)
11–15 years *n* = 9898 (21.0%)	415 (21.2)	0.99 (0.87–1.13)	588 (22.9)	1.15 (1.03–1.28)*	231 (26.6)	1.44 (1.22–1.71)***	29 (25.0)	1.02 (0.84–1.24)	22 (34.9)	2.35 (1.36–4.07)**

Adjusted for age, sex and PHQ-9 score

For 1st five years also adjust for total years in 2nd and 3rd; for 2nd adjust for total in 1st and 3rd; for 3rd adjust for total in 1st and 2nd

^a^Nuclear syndrome excluded from analysis

^b^mean (SD) were used to describe this variable

*P < 0.05, **p < 0.01, ***p < 0.001

Table 3 shows associations between critical timings of exposure to the urban environment at level 3 between birth and 15 years, observed within three time frames of 5 years. Unadjusted findings are not presented in this Table. However, none of the following findings emerged before adjusting for depression: no associations were found for any time periods over the 15 year time span for either paranoid ideation, schizotypal symptoms, or nuclear syndrome. A negative association emerged between psychoticism and critical timings of exposure of between 1 and 3 years during the third 5 year period. Clinical diagnosis of schizophrenia showed strongest association with exposure occurring only during the first 3 years following birth.

**Table 3:** Associations between critical timing of Exposure to the Urban environment between birth and 15 years (*N* = 47,004)

	Psychoticism *n* = 1961 (4.2%)		Paranoia *n* = 2572 (5.5%)		Schizotypal symptoms *n* = 867 (1.8%)^a^		Nuclear syndrome *n* = 116 (0.2%)		Diagnosed Schizophrenia *n* = 63 (0.1%)	
	N (%)	OR (95%CI)	N (%)	OR (95%CI)	N (%)	OR (95%CI)	N (%)	OR (95%CI)	N (%)	OR (95%CI)
**During 1st 5 years (birth - 5 years)**										
0 years (Ref) *n* = 36,548 (77.8%)	1512 (77.1)	Ref (1)	1941 (75.5)	Ref (1)	626 (72.2)	Ref (1)	85 (73.3)	Ref (1)	39 (61.9)	Ref (1)
1–3 years *n* = 810 (1.7%)	50 (2.6)	1.21 (0.81–1.80)	59 (2.3)	1.03 (0.72–1.47)	20 (2.3)	0.88 (0.49–1.56)	3 (2.6)	1.26 (0.29–5.58)	5 (7.9)	7.01 (1.90–25.86)**
4-5 years *n* = 9646 (20.5%)	399 (20.3)	0.92 (0.65–1.31)	572 (22.2)	0.98 (0.72–1.31)	221 (25.5)	0.96 (0.60–1.52)	28 (24.1)	1.27 (0.35–4.64)	19 (31.2)	2.44 (0.55–10.78)
**During 2nd 5 years (6–10 years)**										
0 years (Ref) *n* = 35,400 (75.3%)	1457 (74.3)	Ref (1)	1862 (72.4)	Ref (1)	599 (69.1)	Ref (1)	83 (71.6)	Ref (1)	38 (60.3)	Ref (1)
1–3 years *n* = 984 (2.1%)	58 (3.0)	1.37 (0.91–2.07)	78 (3.0)	1.39 (0.98–1.96)	21 (2.4)	0.67 (0.37–1.21)	4 (3.4)	2.03 (0.53–7.85)	3 (4.8)	2.41 (0.42–13.81)
4-5 years *n* = 10,620 (22.6%)	446 (22.7)	1.28 (0.81–2.02)	632 (24.6)	1.20 (0.82–1.77)	247 (28.5)	1.01 (0.56–1.82)	29 (25.0)	2.01 (0.33–12.17)	22 (34.9)	1.67 (0.20–13.92)
**During 3rd 5 years (11–15 years)**										
0 years (Ref) *n* = 34,021 (72.4%)	1407 (71.8)	Ref (1)	1797 (69.9)	Ref (1)	564 (65.1)	Ref (1)	84 (72.4)	Ref (1)	36 (57.1)	Ref (1)
1–3 years *n* = 1327 (2.8%)	53 (2.7)	0.72 (0.52–1.00)*	68 (2.6)	0.77 (0.58–1.02)	29 (3.3)	1.03 (0.68–1.57)	1 (0.9)	0.14 (0.02–1.15)	3 (4.8)	1.79 (0.52–6.19)
4-5 years *n* = 11,656 (24.8%)	501 (25.5)	1.01 (0.73–1.40)	707 (27.5)	1.11 (0.84–1.47)	274 (31.6)	1.37 (0.90–2.09)	31 (26.7)	0.24 (0.05–1.22)	24 (38.1)	1.57 (0.35–7.06)

Adjusted for age, sex and PHQ-9 score

For 1st five years also adjust for total years in 2nd and 3rd; for 2nd adjust for total in 1st and 3rd; for 3rd adjust for total in 1st and 2nd

^a^Nuclear syndrome excluded from analysis

*P < 0.05, **p < 0.01, ***p < 0.001

### Urbanicity and other risk factors for psychosis

Table 4 shows associations between putative etiological risk factors and the five psychosis phenotypes before and after adjusting for depressive symptoms. All associations showed some degree of attenuation in the table after adjustment, except associations with male sex which became stronger and significant in the case of psychoticism. Following adjustment, associations were no longer significant between psychoticism and ethnic minority status, low family income, and family history of non-psychotic disorder; between paranoid ideation and family history of psychosis; schizotypal symptoms and male sex, family history of psychosis, loss of parent, and sexual abuse; nuclear syndrome and low family income.

**Table 4:** Associations between Etiological risk factors and Psychosis Phenotypes (*N* = 47,004)

	Psychoticism *n* = 1961 (4.2%)		Paranoia *n* = 2572 (5.5%)		Schizotypal symptoms *n* = 867 (1.8%)^a^		Nuclear syndrome *n* = 116 (0.2%)		Diagnosed Schizophrenia *n* = 63 (0.1%)	
	N (%)	OR	N (%)	OR (95%CI)	N (%)	OR (95%CI)	N (%)	OR (95%CI)	N (%)	OR (95%CI)
Male sex *n* = 23,444 (49.9%)	993 (50.6)	1.03 (0.94–1.13)	1253 (48.7)	0.95 (0.88–1.03)	393 (45.3)	0.83 (0.73–0.95)**	59 (50.9)	1.04 (0.72–1.50)	38 (60.3)	1.54 (0.93–2.56)
		1.20 (1.08–1.33)***		1.07 (0.98–1.17)		0.88 (0.76–1.02)		1.06 (0.73–1.55)		1.61 (0.97–2.67)
Ethnic Minority *n* = 4777 (10.2%)	228 (11.6)	1.18 (1.02–1.36)*	278 (10.8)	1.09 (0.96–1.24)	87 (10.0)	1.00 (0.80–1.25)	11 (9.5)	0.91 (0.49–1.70)	9 (14.3)	1.40 (0.69–2.83)
		1.01 (0.86–1.19)		0.94 (0.81–1.09)		0.85 (0.67–1.09)		0.81 (0.42–1.53)		1.30 (0.64–2.64)
Low Family Income *n* = 6986 (14.9%)	361 (18.4)	1.32 (1.17–1.48)***	386 (15.0)	1.03 (0.92–1.15)	135 (15.6)	1.08 (0.90–1.31)	26 (22.4)	1.63 (1.05–2.54)*	23 (36.5)	3.04 (1.81–5.11)***
		1.11 (0.96–1.27)		0.83 (0.73–0.94)**		0.86 (0.70–1.06)		1.32 (0.83–2.10)		2.73 (1.62–4.60)***
Family history psychosis *n* = 338 (0.7%)	32 (1.6)	2.43 (1.68–3.50)***	30 (1.2)	1.68 (1.15–2.45)**	15 (1.7)	2.47 (1.47–4.17)***	1 (0.9)	1.21 (0.17–8.69)	0	–
		1.60 (1.04–2.48)*		1.04 (0.68–1.61)		1.61 (0.89–2.89)		0.74 (0.10–5.55)		–
Family history non-psychotic disorder *n* = 440 (0.9%)	35 (1.9)	2.01 (1.42–2.84)***	52 (2.0)	2.32 (1.73–3.11)***	21 (2.4)	2.66 (1.70–4.14)***	0	–	0	–
		1.25 (0.83–1.89)		1.63 (1.64–2.28)**		1.80 (1.09–2.96)*		–		–
Loss of parent *n* = 632 (1.3%)	46 (2.3)	1.83 (1.35–2.47)***	62 (2.4)	1.91 (1.47–2.49)***	19 (2.2)	1.67 (1.05–2.66)*	1 (0.9)	0.63 (0.09–4.55)	0	–
		1.31 (0.92–1.88)		1.48 (1.09–2.00)*		1.17 (0.70–1.97)		0.43 (0.06–3.18)		–
Physical abuse *n* = 14,785 (31.5%)	1044 (53.2)	2.61 (2.38–2.86)***	1281 (49.8)	2.31 (2.13–2.50)***	464 (53.5)	2.64 (2.30–3.02)***	69 (59.5)	3.23 (2.22–4.68)***	33 (52.4)	2.29 (1.39–3.76)**
		1.75 (1.58–1.95)***		1.62 (1.48–1.77)***		1.69 (1.46–1.96)***		1.88 (1.27–2.77)***		1.71 (1.03–2.85)*
Sexual abuse *n* = 1360 (2.9%)	121 (6.2)	2.43 (1.93–2.84)***	146 (.7)	2.13 (1.79–2.55)***	60 (6.9)	2.52 (1.92–3.29)***	17 (14.7)	5.89 (3.50–9.90)***	3 (4.8)	1.77 (0.55–5.66)
		1.83 (1.09–1.76)**		1.36 (1.10–1.69)**		1.35 (0.98–1.86)		2.63 (1.46–4.75)**		1.01 (0.31–3.35)
Neglect *n* = 16,056 (34.2%)	1183 (60.3)	3.09 (2.81–3.39)***	1487 (57.8)	2.82 (2.60–3.06)***	552 (63.7)	3.50 (3.04–4.02)***	70 (60.3)	2.94 (2.02–4.26)***	37 (58.7)	2.70 (1.64–4.46)***
		1.86 (1.68–2.07)***		1.82 (1.66–1.99)***		2.10 (1.80–2.44)***		1.62 (1.10–2.39)*		1.95 (1.17–3.26)*

Adjusted for age and sex. Second row for each variable further adjusted for PHQ9 score

^a^Nuclear syndrome excluded from analysis

*P < 0.05, **p < 0.01,***p < 0.001

Table 5 shows the associations between other putative risk factors for psychosis (demographic, family history as a proxy for genetic risk, and child maltreatment and disadvantage) and the three measures or urban exposure we found associated with one or more phenotypic expressions of psychosis in Tables 1, 2 and 3. Considering the other risk factors first, male sex was associated with low family income, physical abuse and neglect, and negative associations with family history of non-psychotic disorder and sexual abuse; ethnic minority with low family income, parental loss and neglect; low family income with family history of psychosis, loss of parent, physical abuse and neglect; family history of psychosis with physical abuse and neglect; family history of non-psychotic illness with loss of parent, physical and sexual abuse, and neglect; loss of parent with physical abuse and neglect; physical abuse with sexual abuse and neglect; and sexual abuse showed a negative association with neglect.

**Table 5:** Odds of Co-occurrence of Etiological Risk Factors in Sample

	Male sex	Ethnic Minority	Low Family Income	Family history (psychosis)	Family history (nonpsychotic)	Loss of parent	Physical abuse	Sexual abuse	Neglect
Ethnic Minority	0.92** (0.87–0.98)	\	\	\	\	\	\	\	\
Low Family Income	1.07** (1.02–1.13)	1.68*** (1.56–1.80)	\	\	\	\	\	\	\
Family history (psychosis)	0.93 (0.75–1.15)	1.20 (0.86–1.67)	2.24*** (1.76–2.85)	\	\	\	\	\	\
Family history (nonpsychotic)	0.77** (0.64–0.93)	0.91 (0.66–1.26)	0.76 (0.56–1.01)	–	\	\	\	\	\
Loss of parent	0.96 (0.82–1.13)	1.59*** (1.28–1.97)	2.89*** (2.45–3.42)	1.87 (0.96–3.66)	2.80*** (1.71–4.58)	\	\	\	\
Physical abuse	1.54*** (1.48–1.60)	1.06 (0.99–1.13)	1.14*** (1.08–1.20)	1.38** (1.10–1.72)	1.43*** (1.18–1.74)	1.33*** (1.13–1.56)	\	\	\
Sexual abuse	0.63*** (0.56–0.70)	1.16 (0.98–1.37)	1.01 (0.87–1.18)	1.45 (0.87–2.42)	1.66* (1.09–2.53)	1.15 (0.75–1.75)	1.63*** (1.39–1.91)	\	
Neglect	1.18*** (1.14–1.23)	1.07*** (1.01–1.14)	1.17*** (1.11–1.24)	1.79*** (1.44–2.23)	1.26* (1.03–1.53)	1.46*** (1.24–1.71)	7.19** (6.88–7.52)	0.66*** (0.58–0.75)	\
Urban living 10–15 years	0.90*** (0.86–0.94)	0.74*** (0.69–0.81)	0.28*** (0.26–0.31)	0.77 (0.58–1.02)	1.29* (1.04–1.60)	0.93 (0.76–1.13)	1.03 (0.98–1.08)	0.84* (0.74–0.97)	0.92*** (0.87–0.96)
Urban living Age 1–5 years	0.90*** (0.86–0.94)	0.73*** (0.68–0.79)	0.29*** (0.26–0.32)	0.79 (0.60–1.04)	1.25* (1.01–1.55)	0.89 (0.73–1.08)	1.04 (1.00–1.09)	0.89 (0.78–1.02)	0.93** (0.88–0.97)
Urban birth	0.92** (0.87–0.97)	0.65*** (0.59–0.72)	0.30*** (0.27–0.34)	0.64* (0.44–0.92)	1.22 (0.94–1.57)	0.84 (0.66–1.07)	1.03 (0.97–1.09)	0.84* (0.72–1.00)	0.91*** (0.86–0.96)

*Note*: variable in column is the dependent variable in the regression model

Adjusted for age, sex and PHQ-9 score except for male sex

*P < 0.05, **p < 0.01,***p < 0.001

The three urban exposures tended to show consistency in their associations with other risk factors. Urban living for a total 10–15 years, urban living during 1–5 years of age, and urban birth each showed negative associations with male sex, ethnic minority status, low family income, sexual abuse, and neglect. Urban birth was negatively associated with family history of psychosis and sexual abuse. Urban living for 10–15 years was negatively associated with sexual abuse and positively associated with family history of non-psychotic disorder. Urban living between age 1–5 years was also associated with family history of non-psychotic disorder.

Tables S1–5 show changes to our findings after additionally adjusting for PTSD and dissociative disorder. However, these were small and all trends were in the same direction as before. Overall, this additional sensitivity analysis served to strengthen our conclusions but at the same time was based on wider confidence intervals in our adjusted analyses.

## Discussion

Our study showed that in this student sample urban birth was associated with increased odds of paranoid ideation, schizotypal symptoms, and a clinical diagnosis of schizophrenia. Length of time living in an urban environment had a cumulative effect on schizotypal symptoms, clinical schizophrenia and, to a lesser extent, paranoid ideation. However, this only manifested after 10 years or more of exposure. Only a clinical diagnosis of schizophrenia showed an association with a critical timing of exposure to an urban environment: during early years following birth, between 1 and 3 years. This would suggest that schizophrenia was possibly associated with key urban exposures whilst in utero and soon after birth. However, there was a further effect among those who continued to live in an urban environment for 10 years or more, irrespective of whether they had been born there, possibly suggesting a different type of exposure. Correspondingly, a previous Chinese study of effects on PEs found an interaction between urban birth and time spent in an urban environment.^12^

Similar but increasingly weaker trends were found for effects of urbanicity, including urban birth and length of time living in an urban environment, on schizotypal symptoms, followed by paranoid ideation, but no effects on psychoticism or nuclear syndrome. Our findings therefore suggested a cumulative and dose-response effect of unknown urban exposures acting around the time of birth and influenced by further prolonged exposure to an urban environment. Furthermore, we observed increasing odds of association with increasing severity of the psychosis phenotype, from paranoid ideation, which was not uncommon in the sample, to schizotypal symptoms which were uncommon, to clinical schizophrenia which was rare.

These findings confirmed that associations between urban exposures and psychotic symptoms could be extrapolated to schizophrenia, but these associations only emerged after we had adjusted for depression, dissociative symptoms and PTSD.

### Confounding by depression, dissociative symptoms and PTSD

We are not aware of a previous study which has specifically investigated effects before and after adjustments for symptoms of common mental disorders when investigating associations between urbanicity and psychosis. Significant associations only emerged after statistical adjustments in our models, suggesting our initial findings which found no associations with urbanicity were due to negative confounding from depressive symptoms. Depression is more prevalent among those born in rural areas in China [9–11, 32]. Our initial findings could therefore be explained by most PEs in this student sample being largely associated with depression and not therefore on a continuum with schizophrenia.

Negative associations with male sex showed trend reversal after adjustment. An association with psychoticism became significant. These findings also suggest that initial associations were confounded by depressive symptoms, which are more common in women, and correspond to findings that non-affective psychosis is explained by underlying differences in neurodevelopmental alterations which are more common among men [33].

Although we found significant associations between urbanicity and a clinical diagnosis of schizophrenia, this emerged only following adjustments for depression. Because the prevalence of students reporting a diagnosis of schizophrenia was low, this suggests two possible explanations: firstly, young adults with impaired cognitive abilities who are at increased risk of developing schizophrenia and psychotic conditions with poorer prognosis tend to be excluded from entry to university, or decide not to apply. Secondly, most who experience psychotic symptoms along a continuum with schizophrenia show co-occurring depressive symptoms and may be difficult to differentiate from those along a depression continuum. This form of psychosis in students would present with fewer negative and disorganization symptoms and less developmental impairment [17, 34]. Correspondingly, these same characteristics were identified in earlier clinical research with students with schizophrenia [35].

### What is the urban exposure?

Our findings of co-occurrence of urbanicity and other putative risk factors do not tell us what the urban exposures were which increased the risk for psychosis in this sample. However, they do tell us what they were not. There was no evidence that exposures of urban birth, urban living between 1 and 3 years, or living in a city for 10 years or more were associated with ethnic minority status, low family income, family history of psychosis, experiences of loss of parent, physical abuse, sexual abuse, or neglect – social environmental and proxy genetic factors previously found associated with increased risk of schizophrenia [36]. Although childhood maltreatment was associated with certain psychosis phenotypes in our study, corresponding to previous findings [37], these associations were considerably attenuated following adjustment for depression. Depression is also an outcome of maltreatment [38].

Several other etiological factors we measured were risk factors for both depression and non-affective psychosis. Taking together the associations we observed between psychosis phenotypes and risk factors, and the interrelationships independently observed between the risk factors, these suggest that although social environmental and genetic factors did increase risk of psychosis in this sample, they were not involved in the mechanisms whereby urbanicity increased psychosis risk. Urbanicity and other etiological risk factors therefore operated independently of each other.

It is unclear why a family history of non-psychotic disorder showed associations with both urban living for more than 10 years and urban exposure age 1–3 years, but not with urban birth. However, these effects were relatively weak and the numbers were small. Secondly, this could merely represent a sample effect whereby students who were resident in cities during childhood tended to come from families with higher incomes. The combination of family income and living in an environment with better access to mental health specialists meant that family members with non-psychotic illness were more likely to receive a diagnosis and treatment.

It is also possible that the unusual and somewhat conflicting findings of negative associations in the total sample between urbanicity and family history of psychosis were a generational effect through migration. Psychosis, which has previously been shown to have higher prevalence in Chinese rural areas, would make it less likely for a rural family to migrate successfully to an urban area and find work whilst caring for a psychotic family member. Following adjustment, there were no associations between family history of psychosis and any of the psychosis phenotypes, suggesting confounding by depression. Despite small numbers of students who reported clinical schizophrenia and nuclear syndrome to draw any final conclusions, it was surprising that none reported any family history of either psychotic or non-psychotic mental disorder in the case of schizophrenia, and only one for nuclear syndrome. Associations with other phenotypes showed trends for both family history of psychosis, as expected, together with non-psychotic illness, although these were attenuated following adjustments. It is possible that whatever the urban exposures were, they were somehow related to non-psychotic illness among family members. This could have been the result of psychological stress or some other social environmental factor impacting on the family after moving to a city, prior to or around the participants’ birth rather than a genetic effect. However, it is more likely explained by the aforementioned sample effects, with differing sub-types of psychosis, and where co-associated depression and psychotic symptoms [17, 34] are more predominant among students [35].

### Strengths and limitations

Our large sample with low refusal rate allowed us to test associations with risk factors that were relatively rare. Our sample of students constituted cognitively intact young adults in the age range of early risk for transition from psychotic experiences to clinical psychosis. However, a high-functioning sample of university students meant we had excluded a dimension of important risk factors associated with poor premorbid adjustment, particularly cognitive impairment, more likely to result in negative and disorganisation symptoms in the expression of non-affective psychosis [17, 34]. Nevertheless, we still found categorical, phenotypical expression of psychosis in our sample, corresponding to diagnoses of non-affective psychosis.

One important limitation of the study is the use of SCL-90-R to measure psychosis, particularly in the psychoticism and paranoid ideation phenotypes. Most current research defines PEs as ‘positive’ symptoms of hallucinations, delusions and thought disturbances, whereas the PEs measured using SCL-90-R are mainly based on what would might be classified as schizotypy. Furthermore, several items can be regarded as relational aspects of depression, such as poor self-confidence and somatization/neuroticism. Nevertheless, we did not find associations with nuclear syndrome symptoms despite specifically selecting ‘positive’ items to create this phenotype. Furthermore, adjustment for depressive symptoms is likely to have revealed independent associations with PEs captured by the SCL-90-R.

Other limitations include the use of self-report instruments for all measures of psychopathology. We did not interview participants to confirm whether those with categorical representations of psychosis had actually presented with clinical psychosis. Sample effects and small numbers reporting could explain lack of association between psychosis phenotypes and family history of severe mental disorder.

## Conclusions

We confirmed previously observed associations between urban birth, living 10 years or more in an urban environment, and phenotypic expression of psychosis, demonstrating a hierarchical trend of increasing odds of association from phenotypes of psychotic experiences, to psychotic symptoms, to schizophrenia. Only schizophrenia showed a specific association with urban exposure occurring in the first 5 years following birth. Importantly, none of these findings would have been revealed without adjusting for depression (and in our sensitivity analysis for dissociation and PTSD), indicating that future studies should similarly control for confounding from symptoms of common mental disorders. Whether this new finding in a Chinese sample means that this effect is exclusive to China can only be determined by further study in other countries, particularly developing countries. However, with increasing urbanization and the majority of Chinese now living in urban environments, and with growing evidence that schizophrenia is associated with an urban environment in China [8, 12], our suggest that our study corresponds to previous studies of urbanicity in western countries.

Possible explanations for these findings are that the psychosis phenotypes we investigated in this student sample are associated with two differing domains of etiological risk factors and associated mechanisms: firstly, those which are co-associated with both psychosis and depression but are not on a continuum with schizophrenia. These included genetic loading for both psychotic and non-psychotic disorder, but also stressful life events and poverty which are more prevalent among persons born in rural areas in China [39] and where the phenotypic expression of psychosis overlaps with symptoms of common mental disorder, particularly depression. Secondly, an unknown urban exposure, or exposures, which impact primarily on psychotic and not on depressive symptoms. These are unique to, or have their greatest impact in the urban environment and on persons born in the city. In the case of schizophrenia, those who spent their first years after birth in the city.

The timing of these exposures, together with their level of impact, are more supportive of effects from biological factors in the urban environment rather than either genetic loading or the social environment. These could include effects of factors such as pollution in rapidly industrialising cities [40] or effects on brain development from infections in utero or during infancy [41]. Our findings did not tend to support environment x environment interactions occurring within the process of urbanicity. The overall lack of any associations in this student sample between urbanicity and adverse social environment during childhood was striking. However, such interactions could be involved for co-associated depression and PEs along a depression continuum [42]. Furthermore, our findings did not strongly support gene x environment interactions in this sample. Nevertheless, this possibility should be excluded in further representative, population studies.

## Supplementary Information

**Additional file 1: Table S1**. Associations between Etiological risk factors and Psychosis Phenotypes (*N* = 24,611). **Table S2**. Odds of Co-occurrence of Etiological Risk Factors in Sample. **Table S3**. Associations between Rural-Urban levels of Birth place and Psychosis Phenotypes (*N* = 24,611). **Table S4**. Associations between Length of exposure to an Urban environment and Psychosis Phenotypes (*N* = 24,611). **Table S5**. Associations between critical timing of Exposure to the Urban environment between birth and 15 years (*N* = 24,611). **Table S6**. Prevalence of schizophrenia, depression and mean PHQ-9 scores at different birth level.

## Data Availability

The datasets used and/or analysed during the current study are available from the corresponding author on reasonable request.

## Abbreviations

AOR: Adjusted odds ratio
CI: Confidence interval
PCL-C: PTSD CheckList-Civilian Version
PEs: Psychotic-like experiences
PHQ-9: Patient Health Questionnaire-9
PRIME-MD: Primary Care Evaluation of Mental Disorders
PTSD: Posttraumatic stress disorder
SCL-90-R: Symptom Checklist-90-Revised
SD: Standard deviation

## References

[1] Krabbendam L, van Os J (2005). Schizophrenia and urbanicity: a major environmental influence--conditional on genetic risk. Schizophr Bull.

[2] Marcelis M, Navarro-Mateu F, Murray R, Selten JP, Van Os J (1998). Urbanization and psychosis: a study of 1942-1978 birth cohorts in the Netherlands. Psychol Med.

[3] Stilo SA, Murray RM (2019). Non-genetic factors in schizophrenia. Curr Psychiatry Rep.

[4] Van Os J, Hanssen M, Bijl RV, Vollebergh W (2001). Prevalence of psychotic disorder and community level of psychotic symptoms: an urban-rural comparison. Arch Gen Psychiatry.

[5] Van Os J, Pedersen CB, Mortensen PB (2004). Confirmation of synergy between urbanicity and familial liability in the causation of psychosis. Am J Psychiatry.

[6] Vassos E, Pedersen CB, Murray RM, Collier DA, Lewis CM (2012). Meta-analysis of the association of urbanicity with schizophrenia. Schizophr Bull.

[7] Charlson FJ, Ferrari AJ, Santomauro DF, Diminic S, Stockings E, Scott JG, McGrath JJ, Whiteford HA (2018). Global epidemiology and burden of schizophrenia: findings from the global burden of disease study 2016. Schizophr Bull.

[8] Chan KY, Zhao FF, Meng S, Demaio AR, Reed C, Theodoratou E, Campbell H, Wang W, Rudan I (2015). Urbanization and the prevalence of schizophrenia in China between 1990 and 2010. World Psychiatry.

[9] Huang Y, Wang Y, Wang H, Liu Z, Yu X, Yan J, Yu Y, Kou C, Xu X, Lu J, Wang Z, He S, Xu Y, He Y, Li T, Guo W, Tian H, Xu G, Xu X, Ma Y, Wang L, Wang L, Yan Y, Wang B, Xiao S, Zhou L, Li L, Tan L, Zhang T, Ma C, Li Q, Ding H, Geng H, Jia F, Shi J, Wang S, Zhang N, Du X, Du X, Wu Y (2019). Prevalence of mental disorders in China: a cross-sectional epidemiological study. Lancet Psychiatry.

[10] Phillips MR, Zhang J, Shi Q, Song Z, Ding Z, Pang S, Li X, Zhang Y, Wang Z (2009). Prevalence, treatment, and associated disability of mental disorders in four provinces in China during 2001-05: an epidemiological survey. Lancet.

[11] Chen Y, Bennett D, Clarke R, Guo Y, Yu C, Bian Z, Ma L, Huang Y, Sun Q, Zhang N, Zheng X, Chen J, Peto R, Kendler KS, Li L, Chen Z (2017). Patterns and correlates of major depression in Chinese adults: a cross-sectional study of 0.5 million men and women. Psychol Med.

[12] Chan KY, Zhao FF, Meng S, Demaio AR, Reed C, Theodoratou E, Campbell H, Wang W, Rudan I (2015). Global Health Epidemiology Reference Group (GHERG). Prevalence of schizophrenia in China between 1990 and 2010. J Glob Health.

[13] Coid JW, Hu J, Kallis C, Ping Y, Zhang J, Hu Y, Zhang T, Gonzalez R, Ullrich S, Jones PB, Kirkbride JB (2018). Urban birth, urban living, and work Migrancy: differential effects on psychotic experiences among young Chinese men. Schizophr Bull.

[14] Wang C, Wang Q, Li X, Zhang Y, Wei W, Deng W, Guo W, He L, Tang W, Chen T, Li T (2019). Rural birth/upbringing and childhood adversities are associated with psychotic experiences in university students in China. Schizophr Res.

[15] Koyanagi A, Oh H, Stickley A, Haro JM, DeVylder J (2016). Risk and functional significance of psychotic experiences among individuals with depression in 44 low- and middle-income countries. Psychol Med.

[16] Rössler W, Hengartner MP, Ajdacic-Gross V, Haker H, Gamma A, Angst J (2011). Sub-clinical psychosis symptoms in young adults are risk factors for subsequent common mental disorders. Schizophr Res.

[17] Van Os J, Reininghaus U (2016). Psychosis as a transdiagnostic and extended phenotype in the general population. World Psychiatry.

[18] Varghese D, Scott J, Welham J, Bor W, Najman J, O'Callaghan M, Williams G, McGrath J (2011). Psychotic-like experiences in major depression and anxiety disorders: a population-based survey in young adults. Schizophr Bull.

[19] Verdoux H, van Os J, Maurice-Tison S, Gay B, Salamon R, Bourgeois ML (1999). Increased occurrence of depression in psychosis-prone subjects: a follow-up study in primary care settings. Compr Psychiatry.

[20] Wigman JT, van Nierop M, Vollebergh WA, Lieb R, Beesdo-Baum K, Wittchen HU, van Os J (2012). Evidence that psychotic symptoms are prevalent in disorders of anxiety and depression, impacting on illness onset, risk, and severity--implications for diagnosis and ultra-high risk research. Schizophr Bull.

[21] Guloksuz S, van Nierop M, Lieb R, van Winkel R, Wittchen HU, van Os J (2015). Evidence that the presence of psychosis in non-psychotic disorder is environment-dependent and mediated by severity of non-psychotic psychopathology. Psychol Med.

[22] Van Nierop M, Viechtbauer W, Gunther N, van Zelst C, de Graaf R, Ten Have M, van Dorsselaer S, Bak M, van Winkel R (2015). Childhood trauma is associated with a specific admixture of affective, anxiety, and psychosis symptoms cutting across traditional diagnostic boundaries. Psychol Med.

[23] Mortensen PB, Pedersen CB, Westergaard T, Wohlfahrt J, Ewald H, Mors O, Andersen PK, Melbye M (1999). Effects of family history and place and season of birth on the risk of schizophrenia. N Engl J Med.

[24] Derogatis LR (1977). SCL-90-R: administration, scoring and procedures manual.

[25] Jin H, Wu WW, Zhang MY (1986). Preliminary analysis of SCL90 evaluation results of normal Chinese people. Chinese J Nerv Ment Dis.

[26] Yin BJ, Liu CL (1996). Analysis of EPQ and SCL90 of 2,290 college students. Chin Ment Health J.

[27] Rössler W, Angst J, Gamma A, Haker H, Stulz N, Merikangas KR, Ajdacic-Gross V (2011). Reappraisal of the interplay between psychosis and depression symptoms in the pathogenesis of psychotic syndromes: results from a twenty-year prospective community study. Eur Arch Psychiatry Clin Neurosci.

[28] Kroenke K, Spitzer RL, Williams JB (2001). The PHQ-9: validity of a brief depression severity measure. J Gen Intern Med.

[29] Weathers F, Huska J, Keane T (1991). PTSD Checklist (PCL).

[30] Kessler RC, Ustün TB (2004). The world mental health (WMH) survey initiative version of the World Health Organization (WHO) composite international diagnostic interview (CIDI). Int J Methods Psychiatr Res.

[31] Lee S, Guo WJ, Tsang A, He YL, Huang YQ, Zhang MY, Liu ZR, Shen YC, Kessler RC (2011). The prevalence of family childhood adversities and their association with first onset of DSM-IV disorders in metropolitan China. Psychol Med.

[32] Zhou M, Zhang G, Rozelle S, Kenny K, Xue H (2018). Depressive symptoms of Chinese children: prevalence and correlated factors among subgroups. Int J Environ Res Public Health.

[33] Castle DJ, Murray RM (1991). The neurodevelopmental basis of sex differences in schizophrenia. Psychol Med.

[34] Reininghaus U, Böhnke JR, Hosang G, Farmer A, Burns T, McGuffin P, Bentall RP (2016). Evaluation of the validity and utility of a transdiagnostic psychosis dimension encompassing schizophrenia and bipolar disorder. Br J Psychiatry.

[35] MacCabe JH, Aldouri E, Fahy TA, Sham PC, Murray RM (2002). Do schizophrenic patients who managed to get to university have a non-developmental form of illness?. Psychol Med.

[36] Misiak B, Stramecki F, Gawęda Ł, Prochwicz K, Sąsiadek MM, Moustafa AA, Frydecka D (2018). Interactions between variation in candidate genes and environmental factors in the etiology of schizophrenia and bipolar disorder: a systematic review. Mol Neurobiol.

[37] Varese F, Smeets F, Drukker M, Lieverse R, Lataster T, Viechtbauer W, Read J, van Os J, Bentall RP (2012). Childhood adversities increase the risk of psychosis: a meta-analysis of patient-control, prospective- and cross-sectional cohort studies. Schizophr Bull.

[38] Li M, D'Arcy C, Meng X (2016). Maltreatment in childhood substantially increases the risk of adult depression and anxiety in prospective cohort studies: systematic review, meta-analysis, and proportional attributable fractions. Psychol Med.

[39] Zhang J, Ma Z (2012). Patterns of life events preceding the suicide in rural young Chinese: a case control study. J Affect Disord.

[40] Attademo L, Bernardini F, Garinella R, Compton MT (2017). Environmental pollution and risk of psychotic disorders: a review of the science to date. Schizophr Res.

[41] Khandaker GM, Dalman C, Kappelmann N, Stochl J, Dal H, Kosidou K, Jones PB, Karlsson H (2018). Association of Childhood Infection with IQ and adult nonaffective psychosis in Swedish men: a population-based longitudinal cohort and co-relative study. JAMA Psychiatry.

[42] Coid JW, Zhang Y, Yu H, Li X, Tang W, Wang Q, Deng W, Guo W, Zhao L, Ma X, Meng Y, Li M, Wang H, Chen T, Li T (2021). Confirming diagnostic categories within a depression continuum: testing extra-linearity of risk factors and a latent class analysis. J Affect Disord.

