# Supplementary material for: Impact of urban birth and upbringing on expression of psychosis in a Chinese undergraduate population
Source: BMC Psychiatry. 2021 Oct 9;21:493. doi: 10.1186/s12888-021-03475-w (PMC8501541; doi:10.1186/s12888-021-03475-w)
Supplement: Supplementary file 1 — Additional file 1: Table S1. Associations between Etiological risk factors and Psychosis Phenotypes (N = 24,611). Table S2. Odds of Co-occurrence of Etiological Risk Factors in Sample. Table S3. Associations between Rural-Urban levels of Birth place and Psychosis Phenotypes (N = 24,611). Table S4. Associations between Length of exposure to an Urban environment and Psychosis Phenotypes (N = 24,611). Table S5. Associations between critical timing of Exposure to the Urban environment between birth and 15 years (N = 24,611). Table S6. Prevalence of schizophrenia, depression and mean PHQ-9 scores at different birth level. [file 12888_2021_3475_MOESM1_ESM.docx]

**Supplementary Material**

The following items form Symptom Checklist-90-Revised (SCL-90-R) were used in this study.

**Psychoticism**

7. The idea that someone else can control your thoughts

16. Hearing voices that other people do not hear

35. Other people aware of your private thoughts

62. Having thoughts that are not your own

77. Feeling lonely even when you are with people

84. Having thoughts about sex that bother you a lot

85. The idea that you should be punished for your sins

87. The idea that something serious is wrong with your body

88. Never feeling close to another person

90. The idea that something is wrong with your mind

**Paranoid ideation**

8. Feeling others are to blame for your troubles

18. Feeling that most people cannot be trusted

43. Feeling that you are watched or talked about by others

68. Having ideas or beliefs that others do not share

76. Others not giving you proper credit for achievements

83. Feel that people will take advantage of you if you let them

**Schizophrenia Nuclear Syndrome** was defined based on item 7, 16, 35 and 62;

**Schizotypal symptoms** were defined based on item 8, 18, 43, 68, 76, 77, 83 and 88.

Table S1 Associations between Etiological risk factors and Psychosis Phenotypes (N= 24,611)

|  | Psychoticism  n=1,282 (5.2%) | | Paranoia  n=1,134 (4.6%) | | Schizotypal symptoms  n=637 (2.6%)**^†^** | | Nuclear syndrome  n=78 (0.3%) | | Diagnosed Schizophrenia  n=37 (0.2%) | |
| --- | --- | --- | --- | --- | --- | --- | --- | --- | --- | --- |
|  | N (%) | OR | N (%) | OR (95%CI) | N (%) | OR (95%CI) | N (%) | OR (95%CI) | N (%) | OR (95%CI) |
| Male sex  n=12,546 (50.9) | 654 (5.2) | 1.33*** (1.15-1.53) | 532 (4.2) | 0.99 (0.86-1.14) | 278 (2.2) | 0.84 (0.7-1.02) | 37 (0.3) | 1.02 (0.63-1.63) | 22 (0.2) | 1.54 (0.79-2.98) |
| Ethnic Minority  n=2,602 (10.6) | 143 (5.5) | 1.09 (0.88-1.35) | 125 (4.8) | 1.08 (0.86-1.35) | 55 (2.1) | 0.76 (0.55-1.05) | 9 (0.3) | 1.12 (0.54-2.35) | 7 (0.3) | 1.8 (0.78-4.14) |
| Low Family Income  n=3,311 (13.5) | 239 (7.2) | 1.23* (1.02-1.48) | 186 (5.6) | 0.99 (0.82-1.21) | 107 (3.2) | 0.96 (0.75-1.25) | 19 (0.6) | 1.44 (0.82-2.52) | 13 (0.4) | 2.74** (1.37-5.48) |
| Family history psychosis  n=197 (0.8) | 17 (8.6) | 0.85 (0.45-1.6) | 11 (5.6) | 0.56 (0.27-1.16) | 10 (5.1) | 1.08 (0.5-2.34) | 1 (0.5) | 1.01 (0.13-7.7) | 0 (0) | \ |
| Family history non-psychotic disorder  n=281 (1.1) | 29 (10.3) | 1.15 (0.7-1.88) | 25 (8.9) | 1.09 (0.66-1.8) | 17 (6) | 1.32 (0.72-2.41) | 0 (0) | \ | 0 (0) | \ |
| Loss of parent  n=363 (1.5) | 31 (8.5) | 1.37 (0.86-2.18) | 30 (8.3) | 1.59* (1.01-2.49) | 16 (4.4) | 1.42 (0.79-2.58) | 1 (0.3) | 0.73 (0.1-5.48) | 0 (0) | \ |
| Physical abuse  n=8,785 (35.7) | 8109 (7.7) | 1.25** (1.09-1.44) | 8217 (6.5) | 1.14 (0.99-1.32) | 8444 (3.9) | 1.25* (1.03-1.5) | 8740 (0.5) | 1.32 (0.81-2.15) | 8764 (0.2) | 1.71 (0.87-3.36) |
| Sexual abuse  n=927 (3.8) | 90 (9.7) | 1.58** (1.18-2.12) | 81 (8.7) | 1.56** (1.17-2.09) | 55 (5.9) | 1.85*** (1.3-2.64) | 11 (1.2) | 2.83** (1.40-5.74) | 2 (0.2) | 1.22 (0.29-5.16) |
| Neglect  n=9,381 (38.1) | 781 (8.3) | 1.39*** (1.21-1.6) | 685 (7.3) | 1.40*** (1.22-1.62) | 401 (4.3) | 1.36** (1.13-1.65) | 48 (0.5) | 1.05 (0.63-1.74) | 21 (0.2) | 1.44 (0.73-2.85) |

Adjusted for age, sex, PHQ-9 score, and PCL-C score.

†: Nuclear syndrome excluded from analysis.

*P<0.05, **p<0.01,***p<0.001.

Table S2 Odds of Co-occurrence of Etiological Risk Factors in Sample

|  | Male sex | Ethnic Minority | Low Family Income | Family history (psychosis) | Family history (nonpsychotic) | Loss of parent | Physical abuse | Sexual abuse | Neglect |
| --- | --- | --- | --- | --- | --- | --- | --- | --- | --- |
| Ethnic Minority | 0.82***  (0.75-0.89) | \ | \ | \ | \ | \ | \ | \ | \ |
| Low Family Income | 1.06 (0.99-1.15) | 1.72***  (1.55-1.91) | \ | \ | \ | \ | \ | \ | \ |
| Family history (psychosis) | 0.84  (0.64-1.12) | 1.32  (0.87-1.98) | 1.94***  (1.39-2.71) | \ | \ | \ | \ | \ | \ |
| Family history (nonpsychotic) | 0.57**  (0.45-0.73) | 0.88  (0.59-1.31) | 0.82  (0.56-1.18) | - | \ | \ | \ | \ | \ |
| Loss of parent | 0.90  (0.73-1.11) | 1.42*  (1.06-1.91) | 3.05***  (2.43-3.82) | 0.64  (0.16-2.58) | 2.25*  (1.04-4.03) | \ | \ | \ | \ |
| Physical abuse | 1.63***  (1.55-1.72) | 1.07  (0.98-1.17) | 1.24***  (1.15-1.34) | 1.15  (0.86-1.53) | 1.51***  (1.19-1.93) | 1.17  (0.95-1.46) | \ | \ | \ |
| Sexual abuse | 0.50***  (0.44-0.58) | 1.11  (0.90-1.36) | 1.18  (0.98-1.42) | 1.23  (0.65-2.35) | 1.65*  (1.04-2.63) | 0.90  (0.52-1.58) | 1.81***  (1.47-2.23) | \ |  |
| Neglect | 1.14***  (1.08-1.20) | 1.08  (0.99-1.18) | 1.24***  (1.15-1.34) | 1.56**  (1.17-2.08) | 1.24  (0.97-1.57) | 1.37**  (1.11-1.70) | 8.04*** (7.57-8.54) | 0.54***  (0.46-0.63) | \ |
|  | | | | | | | | | |
| Urban living 10-15 years | 0.80***  (0.75-0.86) | 0.59***  (0.52-0.67) | 0.31***  (0.26-0.36) | 0.65  (0.41-1.03) | 1.26  (0.93-1.71) | 0.70*  (0.50-0.96) | 1.05  (0.98-1.13) | 0.78*  (0.64-0.95) | 0.94***  (0.87-1.01) |
| Urban living Age 1-5 years | 0.80***  (0.75-0.87) | 0.57***  (0.50-0.65) | 0.32***  (0.28-0.37) | 0.63*  (0.41-0.99) | 1.25  (0.93-1.68) | 0.65**  (0.0.47-0.89) | 1.07  (1.00-1.15) | 0.82*  (0.68-0.99) | 0.96  (0.89-1.03) |
| Urban birth | 0.81**  (0.75-0.87) | 0.58***  (0.51-0.66) | 0.31***  (0.27-0.36) | 0.67  (0.43-1.06) | 1.16  (0.85-1.58) | 0.68*  (0.49-0.95) | 1.04  (0.97-1.12) | 0.75**  (0.61-0.91) | 0.94  (0.88-1.01) |

Note: variable in column is the dependent variable in the regression model.

Adjusted for age, sex, PHQ-9 score, and PCL-C score.

†: Nuclear syndrome excluded from analysis.

*P<0.05, **p<0.01,***p<0.001.

Table S3 Associations between Rural-Urban levels of Birth place and Psychosis Phenotypes (N=24,611)

|  | Psychoticism  n=1,282 (5.2%) | | Paranoia  n=1,134 (4.6%) | | Schizotypal symptoms  n=637 (2.6%)**^†^** | | Nuclear syndrome  n=78 (0.3%) | | Diagnosed Schizophrenia  n=37 (0.2%) | |
| --- | --- | --- | --- | --- | --- | --- | --- | --- | --- | --- |
|  | N (%) | OR (95%CI) | N (%) | OR (95%CI) | N (%) | OR (95%CI) | N (%) | OR | N (%) | OR (95%CI) |
| **Birth Exposure** | | | | | | | | | | |
| Level 1 (Rural)  n=12,784 (51.9%) | 761 (5.95) | Ref (1) | 609 (4.76) | Ref (1) | 337 (2.64) | Ref (1) | 42 (0.33) | Ref (1) | 19 (0.15) | Ref (1) |
| Level 2  n=8,022 (32.6%) | 347 (4.33) | 0.86 (0.73-1.01) | 355 (4.43) | 1.19 (1.01-1.39)* | 193 (2.41) | 1.18 (0.96-1.46) | 23 (0.29) | 1.17 (0.68-2.02) | 6 (0.07) | 0.62 (0.24-1.56) |
| Level 3 (Urban)  n=3,805(15.4%) | 174 (4.57) | 0.87 (0.53-1.42) | 170 (4.47) | 0.93 (0.58-1.51) | 107 (2.81) | 1.32 (0.70-2.48)** | 13 (0.34) | 4.01 (0.81-19.95) | 12 (0.32) | 6.68 (0.82-54.74) |

Adjusted for age, sex, PHQ-9 score, and PCL-C score.

†: Nuclear syndrome excluded from analysis.

*P<0.05, **p<0.01, ***p<0.001.

Table S4 Associations between Length of exposure to an Urban environment and Psychosis Phenotypes (N=24,611)

|  | Psychoticism  n=1,282 (5.2%) | | Paranoia  n=1,134 (4.6%) | | Schizotypal symptoms  n=637 (2.6%)**^†^** | | Nuclear syndrome  n=78 (0.3%) | | Diagnosed Schizophrenia  n=37 (0.2%) | |
| --- | --- | --- | --- | --- | --- | --- | --- | --- | --- | --- |
|  | N (%) | OR | N (%) | OR (95%CI) | N (%) | OR (95%CI) | N (%) | OR (95%CI) | N (%) | OR (95%CI) |
| **Urban Exposure** | | | | | | | | | | |
| Total in 15 years^&^  n=24,611 (100%) | 2.49 (5.24) | 1 (0.99-1.02) | 2.75 (5.48) | 1.01 (1-1.03) | 3.00 (5.69) | 1.02 (1.01-1.04)** | 2.69 (5.71) | 1.02 (0.97-1.06) | 4.73 (6.71) | 1.07 (1.02-1.12)** |
| 0 years (Ref)  n=19,088 (77.6%) | 1,010 (5.29) | Ref (1) | 871 (4.56) | Ref(1) | 478 (2.50) | Ref (1) | 63 (0.33) | 0.50 (0.15-1.63) | 24 (0.13) | Ref (1) |
| 1-5 years  n=899 (3.7%) | 46 (5.12) | 0.81 (0.55-1.18) | 45 (5.01) | 0.97 (0.67-1.4) | 27 (3.00) | 1.03 (0.64-1.66) | 1 (0.11) | 0.25 (0.03-1.94) | 0 | \ |
| 6-10 years  n=719 (2.9%) | 43 (5.98) | 1.16 (0.78-1.71) | 37 (5.15) | 1.09 (0.73-1.63) | 20 (2.78) | 0.99 (0.57-1.72) | 0 | \ | 1 (0.14) | 1.13 (0.15-8.47) |
| 11-15 years  n=3,905 (15.9%) | 183 (4.69) | 1.04 (0.85-1.26) | 181 (4.64) | 1.19 (0.98-1.45) | 112 (2.87) | 1.41 (1.11-1.81)** | 14 (0.36) | 1.46 (0.79-2.68) | 12 (0.31) | 2.98 (1.47-6.03)** |

Adjusted for age, sex, PHQ-9 score and PCL-C score.

For 1st five years also adjust for total years in 2nd and 3rd; for 2nd adjust for total in 1st and 3rd; for 3rd adjust for total in 1st and 2nd.

†: Nuclear syndrome excluded from analysis.

^&^mean (SD) were used to describe this variable.

*P<0.05, **p<0.01, ***p<0.001.

Table S5 Associations between critical timing of Exposure to the Urban environment between birth and 15 years (N=24,611)

|  | Psychoticism  n=1,282 (5.2%) | | Paranoia  n=1,134 (4.6%) | | Schizotypal symptoms  n=637 (2.6%)**^†^** | | Nuclear syndrome  n=78 (0.3%) | | Diagnosed Schizophrenia  n=37 (0.2%) | |
| --- | --- | --- | --- | --- | --- | --- | --- | --- | --- | --- |
|  | N (%) | OR (95%CI) | N (%) | OR (95%CI) | N  (%) | OR (95%CI) | N  (%) | OR (95%CI) | N  (%) | OR (95%CI) |
| **During 1st 5 years (birth - 5years)** | | | | | | | | | | |
| 0 years (Ref)  n=20,462 (83.1%) | 1,081 (5.28) | Ref (1) | 941 (4.60) | Ref (1) | 520 (2.54) | Ref (1) | 64 (0.31) | Ref (1) | 24 (0.12) | Ref (1) |
| 1-3years  n=375 (1.5%) | 26 (6.93) | 1.35 (0.74-2.47) | 18 (4.80) | 0.8 (0.41-1.56) | 8 (2.13) | 0.54 (0.21-1.44) | 1 (0.27) | 0.61 (0.06-6.66) | 3 (0.80) | 12.34 (2.76-55.17)** |
| 4-5years  n=3,774 (15.3%) | 175 (4.64) | 1.27 (0.74-2.19) | 175 (4.64) | 1.18 (0.68-2.02) | 109 (2.89) | 1.43 (0.7-2.93) | 13 (0.34) | 1.41 (0.26-7.75) | 10 (0.26) | 7.68 (1.19-49.56)* |
| **During 2nd 5 years (6-10 years)** | | | | | | | | | | |
| 0 years (Ref)  n=19,920 (80.9%) | 1,051 (5.28) | Ref (1) | 911 (4.57) | Ref (1) | 503 (2.53) | Ref (1) | 63 (0.32) | Ref (1) | 25 (0.13) | Ref (1) |
| 1-3years  n=431 (1.8%) | 32 (7.42) | 1.49 (0.83-2.69) | 27 (6.26) | 1.13 (0.61-2.06) | 14 (3.25) | 0.76 (0.33-1.72) | 4 (0.23) | 1.58 (0.17-14.63) | 1 (0.23) | 0.80 (0.06-10.69) |
| 4-5years  n=4,260 (17.3%) | 199 (4.67) | 0.93 (0.46-1.89) | 196 (4.6) | 0.87 (0.44-1.74) | 120 (2.82) | 0.66 (0.27-1.63) | 14 (0.33) | 5.17 (0.55-48.35) | 11 (0.26) | 0.27 (0.01-6.38) |
| **During 3rd 5 years (11-15 years)** | | | | | | | | | | |
| 0 years (Ref)  n=19,309 (78.5%) | 1,023 (5.30) | Ref(1) | 882 (4.57) | Ref (1) | 483 (2.50) | Ref (1) | 64 (0.33) | Ref (1) | 24 (0.12) | Ref (1) |
| 1-3years  n=603 (2.5%) | 26 (4.31) | 1 (0.91-1.1) | 23 (3.81) | 1.03 (0.94-1.13) | 15 (2.49) | 1.06 (0.94-1.19) | 0 | / | 1 (0.17) | 1.19 (0.82-1.72) |
| 4-5years  n=4,699 (19.1%) | 233 (4.96) | 0.94 (0.86-1.02) | 229 (4.87) | 0.89 (0.82-0.97)** | 139 (2.96) | 0.88 (0.79-0.99)* | 14 (0.30) | 0.07 (0.01-0.49)** | 12 (0.26) | 1.55 (1.19-2.03)** |

Adjusted for age, sex, PHQ-9 score and PCL-C score.

For 1st five years also adjust for total years in 2nd and 3rd; for 2nd adjust for total in 1st and 3rd; for 3rd adjust for total in 1st and 2nd.

†: Nuclear syndrome excluded from analysis.

*P<0.05, **p<0.01, ***p<0.001.

Table S6 Prevalence of schizophrenia, depression and mean PHQ-9 scores at different birth level

|  | **Schizophrenia**  **n=63 (0.1%)** | **Depression**  **n=2195(4.67%)** | **PHQ-9 score** |
| --- | --- | --- | --- |
|  | **N (%)** | **N (%)** | **Mean (SD)** |
| Level 1 (Rural)  n=24,479 (52.1%) | 34(0.14) | 1280 (5.23) | 3.22 (3.53) |
| Level 2  n=15,734 (33.5%) | 14(0.09) | 651 (4.14) | 2.81 (3.33) |
| Level 3 (Urban)  n=6791 (14.4%) | 15(0.22) | 264 (3.89) | 2.63 (3.20) |
